# Patient-relevant outcomes following elective, aseptic revision knee arthroplasty: a systematic review

**DOI:** 10.1186/s13643-023-02290-6

**Published:** 2023-08-01

**Authors:** Shiraz A. Sabah, Elizabeth A. Hedge, Lennart von Fritsch, Joshua Xu, Raja Bhaskara Rajasekaran, Thomas W. Hamilton, Alexander D. Shearman, Abtin Alvand, David J. Beard, Sally Hopewell, Andrew J. Price

**Affiliations:** 1 Nuffield Department of Orthopaedics, Rheumatology and Musculoskeletal Sciences, University of Oxford, Oxford, England; 2 Nuffield Orthopaedic Centre, Oxford, England; 3 Centre for Statistics in Medicine, University of Oxford, Oxford, England

**Keywords:** Arthroplasty, Revision/reoperation, Total knee replacement, Patient reported outcome measures, Mortality, Complications

## Abstract

**Background:**

The aim of this systematic review was to summarise the evidence for the clinical effectiveness of revision knee arthroplasty (rKA) compared to non-operative treatment for the management of patients with elective, aseptic causes for a failed knee arthroplasty.

**Methods:**

MEDLINE, Embase, AMED and PsychINFO were searched from inception to 1st December 2020 for studies on patients considering elective, aseptic rKA. Patient-relevant outcomes (PROs) were defined as implant survivorship, joint function, quality of life (QoL), complications and hospital admission impact.

**Results:**

No studies compared elective, aseptic rKA to non-operative management. Forty uncontrolled studies reported on PROs following elective, aseptic rKA (434434 rKA). Pooled estimates for implant survivorship were: 95.5% (95% CI 93.2–97.7%) at 1 year [seven studies (5524 rKA)], 90.8% (95% CI 87.6–94.0%) at 5 years [13 studies (5754 rKA)], 87.4% (95% CI 81.7–93.1%) at 10 years [nine studies (2188 rKA)], and 83.2% (95% CI 76.7–89.7%) at 15 years [two studies (452 rKA)]. Twelve studies (2382 rKA) reported joint function and/or QoL: all found large improvements from baseline to follow-up. Mortality rates were low (0.16% to 2% within 1 year) [four studies (353064 rKA)]. Post-operative complications were common (9.1 to 37.2% at 90 days).

**Conclusion:**

Higher-quality evidence is needed to support patients with decision-making in elective, aseptic rKA. This should include studies comparing operative and non-operative management. Implant survivorship following elective, aseptic rKA was ~ 96% at 1 year, ~ 91% at 5 years and ~ 87% at 10 years. Early complications were common after elective, aseptic rKA and the rates summarised here can be shared with patients during informed consent.

**Systematic review registration:**

PROSPERO CRD42020196922

**Supplementary Information:**

The online version contains supplementary material available at 10.1186/s13643-023-02290-6.

## Background

Knee arthroplasty is a highly successful procedure and, for most patients, is definitive surgery expected to last a lifetime [1]. However, particularly for younger patients, a previously successful knee arthroplasty may deteriorate over time (for example, due to wear of the components) and return to the attention of the surgeon. For other patients, primary knee arthroplasty may have failed to treat the original symptoms. Previous studies have suggested that around 13% of patients are dissatisfied with their outcome following knee arthroplasty [2] and up to 20% of patients have chronic pain [3]. Whilst many of these patients improve with support, those who do not may look to explore revision surgery.

Revision knee arthroplasty (rKA) can be defined as further surgery to an existing knee arthroplasty where a component is added, replaced or modified or the joint is debrided and irrigated [4]. For some patients, there is an absolute indication for rKA, and alternative treatment options are reserved for those unfit (or unwilling) to undergo surgery. This group can include a variety of diagnoses, but urgent indications (such as prosthetic joint infection [PJI] and certain types of fracture) provide unambiguous examples [5]. Elective, aseptic rKA is more common (> 80% cases) [4, 6] and the decision of whether (or when) to undergo rKA follows a shared decision-making process between a patient and their surgeon after discussion of the risks, benefits and alternative treatment options [7]. The goals of surgery in these cases are often similar to primary knee arthroplasty: to reduce pain, improve quality of life and minimise the risk of future complications.

For patients considering elective, aseptic rKA, it follows that full participation in a shared decision-making process requires clear information (supported by high-quality evidence) on the expected outcome should they choose surgery, do nothing or select another type of care [8]. However, the evidence to support these discussions is limited, and has not previously been addressed with a systematic review. As such, the aim of this systematic review was to summarise the evidence for the clinical effectiveness of rKA compared to non-operative treatment for the management of patients with aseptic, non-urgent causes for failed knee arthroplasty.

## Methods

### Patient and public involvement (PPI)

This study is supported by the SORE (Surgery Or REstraint for elective, aseptic revision) knee arthroplasty PPI group and a recent James Lind Alliance Priority Setting Partnership [9].

### Ethics

Research ethics committee (REC) approval was not required for this review.

### Registration and reporting

The study was prospectively registered with PROSPERO (CRD42020196922) and is reported according to the Preferred Reporting Items for Systematic Reviews and Meta-Analyses (PRISMA) 2020 Statement [10]. The completed PRISMA checklist is provided as a Supplementary file.

### Search strategy

Our search strategy (Appendix 1) was designed with an experienced information specialist. MEDLINE, Embase, AMED and PsychINFO were searched from inception to 1st December 2020. There was no restriction on language of publication. Reference lists of included studies were examined to identify further relevant publications.

### Types of study

Randomised and non-randomised studies of patients with a failed rKA treated with elective, aseptic rKA or non-operative management were eligible for inclusion. Randomised studies of any size were eligible for inclusion, whilst non-randomised studies with fewer than 100 patients were excluded due to feasibility.

### Population, Interventions, Comparisons and Outcomes (PICO)

**Fig. 1:**
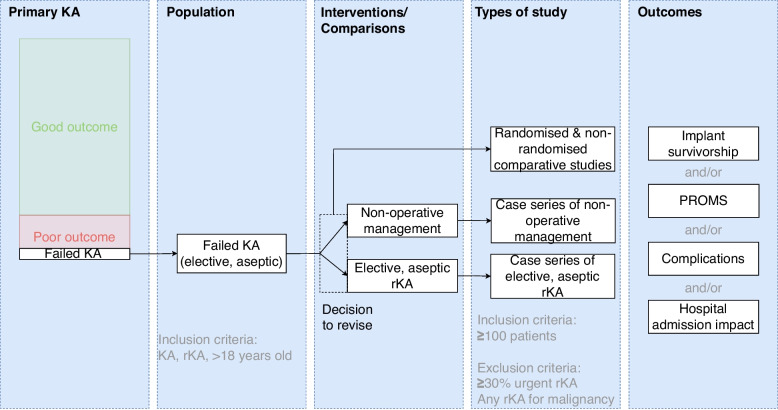
A diagram to illustrate the study population, interventions and comparisons, types of study and patient-relevant outcomes

The PICO framework for this study is illustrated in Fig. 1 and described below.

### Population

Patients aged 18 years or older with a failed KA were eligible for inclusion. A *failed* KA was defined when patients were explicitly stated to be candidates for rKA. We anticipated that this definition may have failed to identify some studies reporting on suitable patients receiving non-operative management. However, we considered it important to be able differentiate this patient group from the larger population with a poor outcome after KA, where revision surgery is often not discussed or offered. We did not consider patient-report outcome measures (PROMs) to be suitable to define failure, since no clear threshold has been defined and current evidence suggests this is likely to vary widely between patients and surgeons [11].

### Interventions and comparators

Revision knee arthroplasty (rKA) was defined as any procedure following primary knee arthroplasty where a component of an arthroplasty was removed, modified, or added [4]. This included isolated exchange of a polyethylene insert, secondary patella resurfacing after total knee arthroplasty, arthroplasty of a further compartment of the knee after partial knee arthroplasty and re-revision surgery. Studies with any procedures for malignancy were excluded. We included studies with up to 30% of procedures for urgent indications (infection or fracture). This threshold was chosen to maximise inclusion of the available literature, without compromising the population of interest. Approximately, 20% of all rKA are performed for ‘urgent’ indications [4, 6]. Non-operative management was defined as any intervention to the joint arthroplasty other than revision arthroplasty (including no treatment).

### Outcomes

The time-points of interest (unless otherwise stated) were defined as: *immediate* (“in-hospital” or up to 30 days), *early* (up to 1 year); *medium-term* (1–5 years); and *longer-term* (over 5 years). Studies were required to report on one or more of the following outcomes:

#### Implant survivorship

The primary outcome of interest was all-cause re-revision surgery (which included both elective, aseptic and non-elective, aseptic reasons for re-revision). Studies were required to report implant survivorship using the Kaplan-Meier method. The time-points of interest were 1, 5, 10 and 15 years. Studies reporting implant survivorship at other time points were rounded down to the nearest of these milestones. A further analysis was performed based on the calculation of person-time incidence rates (PTIRs).

#### Patient-reported outcome measures (PROMs)

‘Joint-specific’ PROMs were defined as instruments addressing one of the following domains: pain, function, combined pain and function, joint-related health status, or patient activity. These instruments were required to be supported by a validation study in a rKA population and to have at least ‘potential for recommendation’ as defined by the COnsensus-based Standards for the selection of health status Measurement INstruments (COSMIN) initiative [12]. The Knee Injury and Osteoarthritis Outcome Score (KOOS) [13], Lower Extremity Activity Scale (LEAS) [14], Oxford Knee Score (OKS) [15, 16], and Western Ontario and McMaster Universities Arthritis Index (WOMAC) [17] instruments met these criteria. A clinically meaningful change following elective, aseptic rKA has only been defined for the OKS (where the MIC_group_ = 9.5 points) [16, 18]. For health-related quality of life (QoL) and anxiety or depression we did not require instruments to have been validated specifically for elective, aseptic rKA.

#### Acquired comorbidity (including mortality)

Acquired comorbidity following rKA was recorded for immediate and early follow-up. The following adverse events were recorded: death, allogeneic blood transfusion, cardiac complications, central nervous system complications, deep vein thrombosis, pulmonary embolism, genitourinary complications, renal complications, respiratory complications, post-operative infection (such as deep surgical site infection or sepsis) and wound dehiscence. We also recorded the incidence of ‘any complication’ where reported as such in a study. This system was chosen based on prior knowledge of World Health Organisation (WHO) International Classification of Disease (ICD) codes, which it was anticipated that many studies would use [19].

#### Hospital admission impact

Hospital admission impact was evaluated according to length of stay, requirement for high-dependency or intensive care, and hospital re-admission.

### Data extraction and management

All citations were imported to the web application Rayyan [20]. De-duplication and abstract screening was performed by two review authors (SS and JX/LF). The full-text of each study potentially meeting inclusion criteria was screened by two reviewers (SS and AS/EH/RB/TH). Disagreements were resolved through discussion. A standardised data collection form was created using the Research Electronic Data Capture (REDCap) data management platform and piloted to ensure consistency and ease of use [21]. Data were extracted on study design, dates of study, number of sites and location, and study setting. Participant enrolment and withdrawals were recorded, together with demographic information (age, gender, comorbidities and revision diagnosis). The funding source and notable declarations of interest for trial authors were recorded. Data were extracted from figures at the discretion of the lead author.

### Data analysis

Meta-analysis was performed for implant survivorship at 1, 5, 10 and 15 years following assessment of clinical and methodological homogeneity. The included studies were required to report survivorship using Kaplan-Meier estimates, under the assumption that these estimates approximated risk. The Stata package *metan* was used for analysis. A random effects model was used to account for variability among the included studies (for example, due to different characteristics of the patient groups). Statistical heterogeneity was assessed by visual inspection of the forest plot for obvious differences in results between the studies, and by using the *I*^2^ and chi^2^ statistical tests. Where studies did not report a 95% confidence interval around the Kaplan-Meier estimate, simple imputation was performed to impute the mean standard error calculated from the other studies reporting at that time point. A sensitivity analysis was performed to examine the effect of excluding studies with imputed data. Since not all studies reported Kaplan-Meier estimates, an additional analysis was performed for studies that provided data where person time incidence rates (PTIRs) could be calculated. The denominator for rate was calculated by multiplying the number of patients with the mean follow-up. The numerator was calculated by totalling the number of first re-revisions over the study follow-up. The PTIR was then expressed as the number of re-revisions per 100 patient years at risk (which corresponds with current NJR methodology) [22]. Secondary outcome measures (patient reported outcome measures, acquired comorbidity, and hospital admission impact) were evaluated using narrative synthesis with results organised into tables.

### Quality assessment

Two authors (SS and EH/RB) independently assessed study quality according to the checklist proposed by Wylde et al. [23], which was designed for studies on joint arthroplasty. The tool evaluates bias due to patient selection (two items), missing data (one item) and confounding (one item). Each item is rated either ‘adequate’ or ‘inadequate’ and reported individually, rather than as a summary score. An adequate rating is given to (i) recruitment of consecutive patients, (ii) recruitment of patients from multiple centres, (iii) follow-up of more than 80% of patients and (iv) use of a multivariable model.

### Missing data

We did not contact investigators or study sponsors to obtain missing outcome data.

### Software

Statistical analyses were performed using Stata (StataCorp. 2019. Stata Statistical Software: Release 16. College Station, TX: StataCorp LLC.)

## Results

**Fig. 2:**
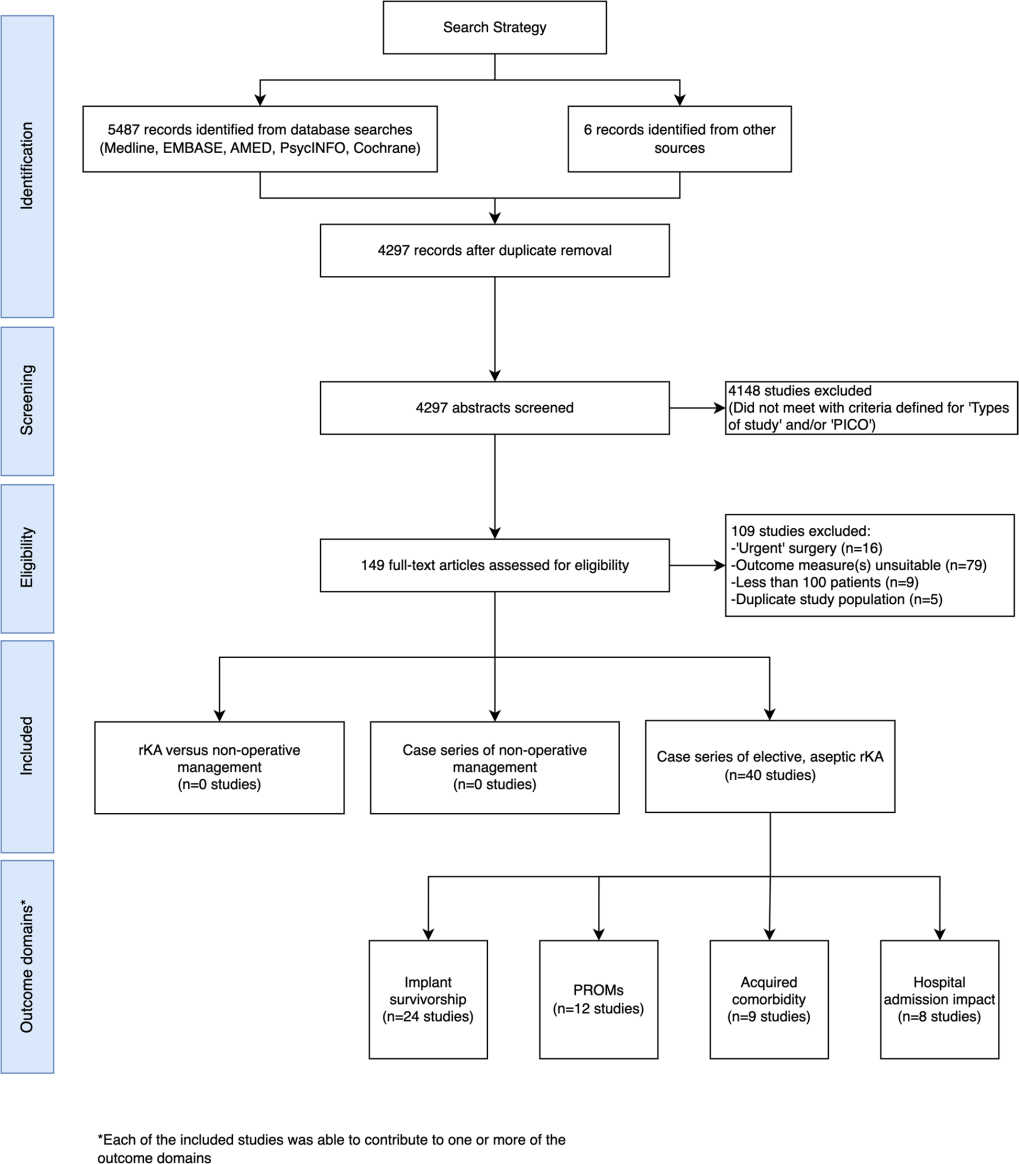
PRISMA flow diagram

After deduplication, the titles and abstracts were screened for 4297 articles. 149 full-text articles were assessed for eligibility. The PRISMA flow diagram is provided in Fig. 2. No randomised or non-randomised studies were identified that reported on patient-relevant outcomes following elective, aseptic rKA compared to another form of care. No studies reported on patient-relevant outcomes following non-operative management for failed KA. Forty non-randomised, uncontrolled studies (434,434 rKA) [24–63] reported on patient-relevant outcomes following elective, aseptic rKA and were included in this review (Table 1).

**Table 1:** Overview of included studies

						Domains of patient-relevant outcomes			
Study^a^	Study design	Brief aim *(To investigate…)*	No. rKA	Female (%)	Age in years *(mean (sd))*	Implant survivorship	PROMs	Acquired comorbidity	Hospital admission impact
Bloch et al. (2020) [24]	Retrospective observational (single centre)	Implant survivorship of metaphyseal sleeves	316	52	70 (10)	Yes			
Dai et al. (2020) [35]	Retrospective observational (NIS)	Immediate complications of rKA	5187	51	66 (NS)			Yes	
Martin et al. (2020) [46]	Retrospective observational (single centre)	Implant survivorship following rKA for aseptic loosening tibia	164	62	median 64 (Q1-Q3 59–71)	Yes			
Piuzzi et al. (2020) [57]	Prospective cohort (OME)	Joint function after aseptic rKA	246	57	65 (10)		Yes		
Bin Abd Razak et al. (2019) [59]	Retrospective observational (single centre)	Joint function after rKA	163	77	68 (NS)	Yes	Yes		Yes
Edmiston et al (2019) [60]	Retrospective observational (CCAE/MDCR)	Impact of patient comorbidity on surgical site infection	14486	58	66 (11)			Yes	
Sachdeva et al. (2019) [61]	Retrospective observational (single centre)	Implant survivorship and joint function after aseptic rKA	100	64	64 (NS)	Yes			
Stevens et al. (2019) [62]	Retrospective observational (single centre)	Implant survivorship and joint function after rKA	100	58	70 (10)	Yes	Yes		
Stockwell et al. (2019) [63]	Retrospective observational (single centre)	Implant survivorship and joint function after rKA	170	57	68 (NS)	Yes	Yes		
Turnbull et al. (2019) [25]	Retrospective observational (single centre)	Joint function after rKA	112	44	71 (10)	Yes	Yes		
Yao et al. (2019) [26]	Retrospective observational (single centre)	Mortality after rKA	3138	53	68 (11)			Yes	
Boddapati et al. (2018) [27]	Retrospective, observational (NSQIP)	Immediate complications and hospital admission impact of rKA (aseptic versus PJI)	10584	60	NS			Yes	Yes
Lombardi et al. (2018) [28]	Retrospective observational (single centre)	Implant survivorship following rKA for failed unicompartmental KA	193	60	64 (NS)	Yes			
Boylan et al. (2017) [29]	Retrospective observational (SPARCS)	Venous thromboembolism after rKA	16630	61	66 (NS)			Yes	
Burnett et al. (2017) [30]	Retrospective observational (Humana)	Blood transfusion after rKA	12493	61	NS			Yes	
Crawford et al. (2017) [31]	Retrospective observational (single centre)	Implant survivorship after aseptic rKA usaing modular system	278	60	67 (NS)	Yes			
Kim et al. (2017) [32]	Retrospective observational (multicentre)	Clinical outcomes following mobile-bearing rKA	280	58	66 (NS)	Yes			
Liang et al. (2017) [33]	Retrospective observational (single centre)	Implant survivorship and mode of failure for rKA	258	92	66 (10)	Yes			
Martin-Hernandez et al. (2017) [34]	Prospective cohort (single centre)	Joint function after rKA using metaphyseal sleeves	134	61	Median 75 (range 51–88)		Yes		
Siqueira et al. (2017) [36]	Retrospective observational (single centre)	Implant survivorship of varus-valgus constrained aseptic rKA	315	59	66 (12)	Yes			
Bini et al. (2016) [37]	Retrospective observational (TJRR)	Implant survivorship of aseptic rKA	1154	61	65 (10)	Yes			
Leta et al. (2016) [38]	Retrospective observational (NAR)	Implant survivorship and joint function following secondary patella resurfacing	308	73	NS	Yes	Yes		
Nichols et al. (2016) [39]	Retrospective, observational (MarketScan)	Immediate complications of rKA	25354	58	63 (11)			Yes	Yes
Graichen et al. (2015) [40]	Retrospective observational (single centre)	Implant survivorship after aseptic rKA using metaphyseal sleeves	121	69	74 (9)	Yes			
Kim et al. (2015) [41]	Retrospective observational (single centre)	Clinical outcomes after condylar constrained rKA	228	87	65 (10)	Yes	Yes		Yes
Kasmire et al. (2014) [42]	Retrospective observational (single centre)	Joint function after aseptic rKA	175	63	66 (NS)		Yes		Yes
Kremers et al. (2014) [43]	Retrospective observational (single centre)	The effect of obesity on medical costs in KA	1654	53	NS			Yes	Yes
Schairer et al. (2014) [44]	Retrospective observational (single centre)	Hospital readmission after rKA	262	56	62 (13)				Yes
Sierra et al. (2013) [45]	Retrospective observational (multicentre)	Implant survivorship following rKA for failed unicompartmental KA	175	52	66 (NS)	Yes			
Venkataramanan et al. (2013) [47]	Retrospective observational (multicentre)	Patient-reported outcomes after rKA	145	54	69 (10)		Yes		
Baker et al. (2012) [48]	Retrospective observational (NJR-PROMs)	Patient-reported outcomes by diagnosis after aseptic rKA	797	53	68 (10)		Yes		
Engh et al. (2012) [49]	Retrospective observational (single centre)	Implant survivorship after rKA for polyethylene wear	119	45	68 (NS)	Yes			
Hardeman et al. (2012) [50]	Retrospective observational (single centre)	Implant survivorship after rKA	146	NS	68 (NS)	Yes			
Malviya et al. (2012) [51]	Retrospective observational (single centre)	Joint function after rKA	120	53	69 (NS)		Yes		
Ong et al. (2010) [52]	Retrospective observational (Medicare)	Implant survivorship after rKA	1599	63	72 (5)	Yes			
Wood et al. (2009) [53]	Retrospective observational (single centre)	Implant survivorship after rKA using press-fit stem	135	56	71 (NS)	Yes			
Memtsoudis et al. (2008) [54]	Retrospective, observational (NHDS)	Immediate complications of rKA	334155	58	68 (NS)			Yes	Yes
Suarez et al. (2008) [55]	Retrospective observational (single centre)	Implant survivorship after rKA	443	NS	66 (NS)	Yes			
Sheng et al. (2006) [56]	Retrospective observational (FAR)	Implant survivorship after first rKA	1874	NS	69 (NS)	Yes			
Bugbee et al. (2001) [58]	Retrospective observational (single centre)	Implant survivorship after rKA	123	NS	NS	Yes			

*CCAE* IBM Market Scan Commercial Claims and Encounters (CCAE), *MDCR* Medicare Supplemental and Coordination of Benefits, *FAR* Finnish arthroplasty registry, *NHDS* National Hospital Discharge Survey, *NIS* Nationwide Inpatient Sample, *NJR-PROMs* National Joint Registry (UK) linked to NHS Patient Reported Outcome Measures dataset, *NS* not specified, *NSQIP* American College of Surgeons National Surgical Quality Improvement Program, *rKA* revision total knee arthroplasty, *SPARCS* New York Statewide Planning and Research Cooperative System database

^a^Sorted by year (most recent at top)

### Outcome measures

#### Implant survivorship

**Fig. 3:**
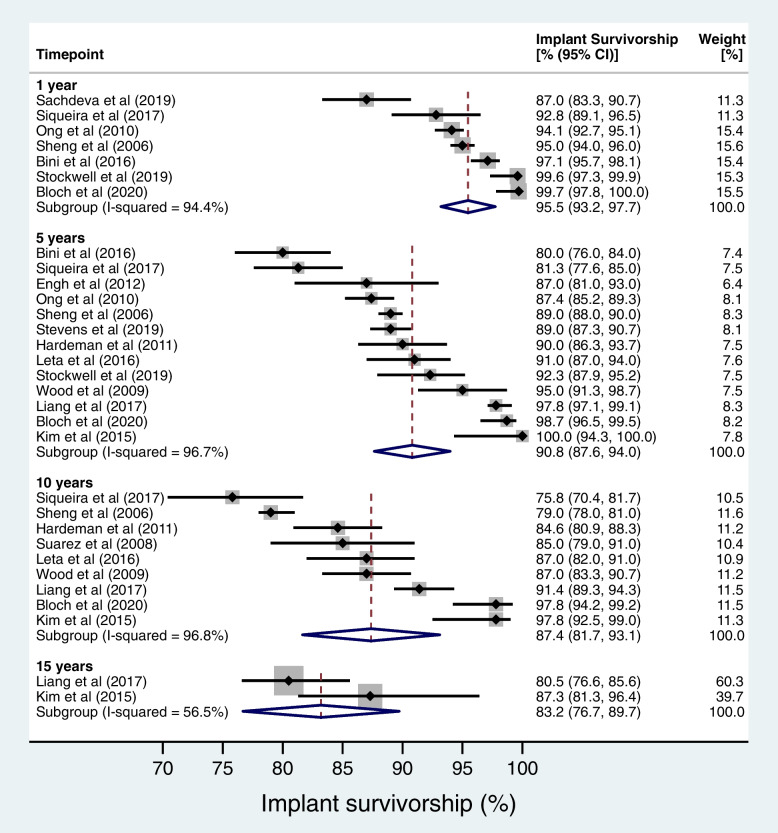
Forest plot of estimates for reported implant survivorship following elective, aseptic rKA

Fifteen studies [24, 33, 36–38, 41, 49, 50, 52, 53, 55, 56, 61–63] reported all-cause implant survivorship for 7227 rKA (Fig. 3). Seven studies (5524 rKA) reported survivorship at 1 year, 13 studies (5,754 rKA) at 5 years, nine studies (2188 rKA) at 10 years and two studies (452 rKA) at 15 years. Pooled analysis of data found all-cause implant survivorship of 95.5% (95% CI 93.2–97.7%) at 1 year, 90.8% (95% CI 87.6–94.0%) at 5 years, 87.4% (95% CI 81.7-93.1%) at 10-years, and 83.2% (95% CI 76.7–89.7%) at 15 years. These estimates changed little when studies that did not report confidence intervals for survivorship estimates were excluded (Appendix 2 Figure 1). Eighteen studies (3205 rKA) [24, 25, 28, 31–33, 40, 41, 45, 46, 49, 50, 53, 58, 59, 61–63] provided data from which person-time incidence rates could be calculated. These are provided as a further sensitivity analysis in Appendix 2 Table 1.

#### Patient-reported outcome measures (PROMs)

Twelve studies [25, 34, 38, 41, 42, 47, 48, 51, 57, 59, 62, 63] reported on the outcome of 2382 rKA with one or more returned PROM questionnaires (Appendix 3 Table 1). The instruments used to report joint function were: KOOS (2 studies), OKS (5 studies), and WOMAC (5 studies). The instruments used to report QoL were EQ-5D (2 studies), SF-12 (2 studies) and SF-36 (3 studies). None of the included studies measured anxiety or depression using dedicated instruments, though these domains were assessed within some of PROMs listed above. Ten of the 12 studies (83.3%) reporting on joint-function and 6 of the 7 studies (85.7%) reporting on QoL provided both pre-operative and post-operative summary statistics. Each of these studies reported improvement in joint-function and QoL following elective, aseptic rKA. Indeed, the two studies that reported mean change in score using the OKS [48, 59], both found that improvement in joint function exceeded the MICgroup estimate of 9.5 points at all post-operative timepoints.

#### Acquired comorbidity

##### Mortality

Four studies (353,064 rKA) reported mortality rates after rKA [26, 27, 35, 54] (Table 2). Three studies reported on *immediate-term* mortality [27, 35, 54] with estimates ranging from 0.16 to 0.30%. Yao et al [26] reported an *early* (1 year) mortality rate of 1–2% for indications other than fracture and infection from a single tertiary centre in the USA between 1985 and 2015.

**Table 2:** Studies reporting on mortality after rKA

Study^a^	Study design	Timepoint	No. rKA	No. Deaths	Mortality rate
Memtsoudis et al. (2008) [54]	Retrospective observational (NHDS)	Immediate (“in hospital”)	33,4155	560	0.20%
Dai et al. (2020) [35]	Retrospective observational (NIS)	Immediate (“in hospital”)	5187	14	0.30%
Boddapati et al. (2017) [27]	Retrospective observational (NSQIP)	Immediate (30 days)	10,584	NS	0.16%
Yao et al. (2018) [26]	Retrospective observational (single centre)	Early (1 year)	3138	NS	1-2%^b^

*NS* not specified, *NHDS* National Hospital Discharge Survey, *NIS* Nationwide Inpatient Sample, *NSQIP* American College of Surgeons National Surgical Quality Improvement Program, *rKA* revision total knee arthroplasty

^a^Sorted by timepoint of assessment, then study size

^b^For indications other than fracture and infection

##### Blood transfusion

Four studies [27, 30, 35, 39] (53,618 rKA) reported on the need for blood transfusion following rKA (Appendix 4 Table 1). All studies were based in the USA and the rate of blood transfusion ranged from 8.4% [39] to 18.4% [35]. Nichols et al. [39] analysed the Marketscan administrative claims dataset and reported a rate of allogeneic blood transfusion of 7.9% during the index hospitalisation, with a further 0.5% requiring autologous blood transfusion. Dai et al. [35] reported a transfusion rate of 18.4% during the index hospitalisation from 5187 patients within the US Nationwide Inpatient Sample (NIS). Burnett et al [30] analysed the Humana Inc. administrative claims database where 11.9% of patients required blood transfusion within 3 days of rKA between 2007 and 2015. Most transfusions (92.0%) were with allogeneic packed red blood cells and they found a 72% reduction in requirement for blood transfusion from 2007 (15.9% rKA) to 2015 (4.5% rKA). Boddapati et al [27] analysed data from the American College of Surgeons National Surgical Quality Improvement Program (ACS-NSQIP) where they found a transfusion rate of 11.9% within 30 days of aseptic rKA.

##### Complications

Seven studies [27, 29, 35, 39, 43, 54, 60] (408,050 rKA) reported on complications after rKA (Table 3). Three studies reported immediate complications [27, 35, 54] and four studies reported early complications [29, 39, 43, 60]. The rate of any complication at 90 days ranged from 9.1 [43] to 37.2% [39]. The reported rate of surgical site infection ranged from 15.6 [60] to 24.1% [39] in the two studies reporting early complications. Studies reporting immediate complications all reported lower rates of post-operative infection (< 1%). The specific complication of wound dehiscence was identified in 0.3% [54] to 1.7% [39] rKA. Medical complications included: deep vein thrombosis (0.2% [35] to 1.7% [29] rKA), pulmonary embolism (0.1% [39] to 0.6% [29] rKA), cardiac complications (0.3% [39] to 0.9% [54] rKA) and central nervous system complications (0.1% [27, 54] rKA).

**Table 3:** Early complications after rKA

Study^a^	Study design	Timepoint	No. rKA	Any complication^b^	Cardiac^b^	Central nervous system^b^	Deep vein thrombosis^b^	Pulmonary embolism^b^	Genitourinary^b^	Renal^b^	Respiratory^b^	Post-operative infection^b^	Wound dehiscence^b^
Memtsoudis et al. (2008) [54]	Retrospective observational (NHDS)	Immediate (“in hospital”)	334,155	29,007 (8.7%)	3141 (0.9%)	308 (0.1%)	NS	NS	3020 (1%)	NS	4393 (1.3%)	2241 (0.7)	854 (0.3%)
Dai et al. (2020) [35]	Retrospective observational (NIS)	Immediate (“in hospital”)	5187	1025 (19.8%)	41 (0.8%)	NS	10 (0.2%)	12 (0.2%)	32 (0.6%)	NS	37 (0.7%)	43 (0.8%)	27 (0.5%)
Boddapati et al. (2017) [27]^c^	Retrospective observational (NSQIP)	Immediate (30 days)	10,584	(4.7%)	(0.4%)	(0.1%)	(0.9%)	NS	(0.9%)	(0.3%)	(0.6%)	(0.6%)^d^	40 (0.4%)
Nichols et al. (2016) [39]^c^	Retrospective observational (MarketScan)	Early (90 days)	25,354	(37.2%)	(0.3%)	NS	NS	(0.1%)	NS	(0.2%)	(1.3%)	(24.1%)	(1.7%)
Boylan et al. (2017) [29]	Retrospective observational (SPARCS)	Early (90 days)	16,630	NS	NS	NS	276 (1.7%)	105 (0.6%)	NS	NS	NS	NS	NS
Edmiston et al. (2019) [60]	Retrospective observational (CCAE/MDCR)	Early (90 days)	14,486	NS	NS	NS	NS	NS	NS	NS	NS	2259 (15.6%)	NS
Kremers et al. (2014) [43]	Retrospective observational (single centre)	Early (90 days)	1654	151 (9.1%)	NS	NS	NS	NS	NS	NS	NS	NS	NS

*CCAE* IBM Market Scan Commercial Claims and Encounters (CCAE), *MDCR* Medicare Supplemental and Coordination of Benefits, *NHDS* National Hospital Discharge Survey, *NIS* Nationwide Inpatient Sample, *NS* not specified, *NSQIP* American College of Surgeons National Surgical Quality Improvement Program, *rKA* revision total knee arthroplasty, *SPARCS* New York Statewide Planning and Research Cooperative System database

^a^Sorted by timepoint of assessment, then study size

^b^*n* (%)

^c^Study reported percentage frequency experiencing outcome only

^d^Coded separately as deep surgical site infection/sepsis after rKA for aseptic indications

#### Hospital admission impact

##### Length of stay (LOS)

Eight studies [27, 39, 41–44, 54, 59] (372575 rKA) reported on LOS after rKA (Table 4). Among the studies based in the USA mean LOS ranged from 3.4 days [27] to 5.6 days [39]. Bin Abd Razak et al [59] reported a mean LOS of 7.7 days at a single tertiary centre in Singapore. Whilst Kim et al [41] reported a mean LOS of 16 days following rKA in the Republic of Korea from a single surgeon series.

**Table 4:** Studies reporting on length of stay (LOS) after rKA

Study^a^	Study design	No. rKA	Mean LOS/days	SD LOS /days
Memtsoudis et al. (2008) [54]	Retrospective observational (NHDS)	33,4155	5.4	NS
Nichols et al. (2016) [39]	Retrospective observational (MarketScan)	25,354	5.6	7.2
Boddapati et al. (2017) [27]	Retrospective observational (NSQIP)	10,584	3.4	3.3
Kremers et al. (2014) [43]	Retrospective observational (single centre)	1654	5.3	3.1
Schairer et al. (2014) [44]	Retrospective observational (single centre)	262	4.6	2.5
Kim et al. (2015) [41]	Retrospective observational (single centre)	228	16	NS
Kasmire et al. (2014) [42]	Retrospective observational (single centre)	175	4.3	NS
Bin Abd Razak et al. (2019) [59]	Retrospective observational (single centre)	163	7.7	NS

*NHDS* National Hospital Discharge Survey, *NS* not specified, *NSQIP* American College of Surgeons National Surgical Quality Improvement Program, *rKA* revision total knee arthroplasty

^a^Sorted by study size

##### High-dependency care

None of the included studies provided information on high-dependency care utilisation after rKA.

##### Hospital re-admission

Three studies (36,200 rKA), all from the United States, reported on hospital re-admission after rKA [27, 39, 44] (Table 5). Boddapati et al [27] analysed data from 10584 aseptic rKA within ACS-NSQIP between 2005 and 2015 where they identified a readmission rate of 6% at 30 days. Nichols et al [39] reported a 23% re-admission rate at 90 days based on data from 25,354 rKA registered with the Truven MarketScan database in North America from 2009 to 2013. Schairer et al. [44] reported a 13% re-admission rate at 90 days using a hospital administrative claims database of 262 rKA from 2005 to 2011.

**Table 5:** Studies reporting on hospital re-admission after rKA

Study^a^	Study design	Timepoint	No. rKA	Readmissions *n* (%)
Boddapati et al. (2017) [27]	Retrospective observational (NSQIP)	Immediate (30 days)	10,584	581 (5.5)
Nichols et al. (2016) [39]	Retrospective observational (MarketScan)	Early (90 days)	25,354	5857 (23.1)
Schairer et al. (2014) [44]	Retrospective observational (single centre)	Early (90 days)	262	34 (13.0)

*NSQIP* American College of Surgeons National Surgical Quality Improvement Program, *rKA* revision total knee arthroplasty

^a^Sorted by timepoint of assessment, then study size

### Quality assessment

Among the 40 studies included, 21 studies (53%) recruited consecutive patients, 15 studies (38%) were multicentre, 31 studies (78%) had adequate patient follow-up and 20 studies (50%) included a multivariable regression model (Appendix 5 Table 1).

## Discussion

This study has summarised patient-relevant outcomes (PROs) following elective, aseptic revision knee arthroplasty (rKA). The quality of the included studies was low, comprising uncontrolled observational series. We did not find any studies comparing PROs following revision surgery to non-operative management or no treatment at all. We have addressed the question: “How long is an elective, aseptic rKA expected to last?”. We found rKA survivorship ~ 96% at 1 year, ~91% at 5 years, ~87% at 10 years and ~ 83% at 15 years. All studies reporting on joint function and quality-of-life showed large improvements at early timepoints following rKA. We also reported the rate of complications following elective, aseptic rKA. These estimates may be useful to support the process of informed consent. The risk of death in the immediate post-operative period was low, with reported rates of 0.16% to 0.30%. Only one study reported mortality at 1 year, with a rate of 2%. The rate of any complication was highly variable (from 9.1 to 37.2% at 90 days following surgery). This is likely to reflect the heterogeneity of both patients undergoing elective, aseptic rKA and the procedures themselves. Post-operative infection (which is a set of administrative codes incorporating both systemic sepsis and surgical site infections) was one of the most common complications. There were large differences between studies reporting rates at immediate timepoints (< 1% “in-hospital” or within 30 days) compared to those reporting at early timepoints (15.6% to 24.1% within 90 days). Cardiac, central nervous system, genitourinary, renal and respiratory complications were all rare (~ 1% or less at 90 days). The rate of deep vein thrombosis ranged from 0.2 to 1.7%, while the rate of pulmonary embolism ranged from 0.1 to 0.6% at 90 days. With respect to the hospital admission, the mean length of stay (LOS) in the USA ranged from 3.4 days to 5.6 days. The two studies included from Singapore and Korea both reported longer mean LOS. We have not explored the reasons for this. The rate of re-admission to hospital ranged from 13 to 23% within the first 90 days. Patients undergoing elective, aseptic rKA were at high risk for blood transfusion, with rates of 8.4 to 18.4% reported.

A number of relevant studies have been published since the literature search for this review was completed. Deere et al. [64] reported on implant survivorship following first and multiple rKA procedures using data from the National Joint Registry (NJR) for England, Wales, Northern Ireland, the Isle of Man and the States of Guernsey. They reported Kaplan Meier survivorship estimates for first rKA procedures of 96.4% at 1 year, 87.4% at 5 years and 82.9% at 10 years. The reported re-revision rates at 5- and 10 years were higher than in the present study, which may be due to the inclusion of ‘urgent’ rKA procedures. They found that male gender and younger age were risk factors for multiple revisions. A recent study from our group reported on mortality and complication rates following 30,826 elective rKA procedures recorded in Hospital Episode Statistics (HES) in the UK [5]. This found a 90-day mortality rate of 0.44%, which is comparable to the estimates reported in this review, and similar to primary KA (0.46%). Of note, the early mortality rate following infected rKA appears to be greater (2.04% at 90 days) [5]. A further study from our group reported on patient-reported outcome measures following elective, aseptic rKA in 10,727 patients from the NHS PROMs dataset [11]. This found that two-thirds of patients experienced a meaningful improvement in joint function after rTKA, 69.4% were satisfied with the procedure and 74.1% felt that surgery was a success [11]. However, the rate of early patient-reported complications was very high (46.0% at 6 months)—which is much higher than reported in administrative datasets, as confirmed by the current review—and this finding requires further exploration.

A major strength of this study is that we have reported domains of outcomes following surgery that patients themselves have identified to be important [65]. Whilst the quality of the included studies was low, we predicted this with the design of our review. Due to the preponderance of small, low-quality studies reporting on elective, aseptic rKA, one inclusion criterion we enforced (based on feasibility) was to exclude studies with fewer than 100 participants. This has resulted in bias towards larger studies (such as those reporting data from joint registries and other routine healthcare datasets). On the one hand, these studies have enabled us to capture data on rare outcomes (such as mortality and a range of different complications). However, the limitations of administrative data coding and the restricted perspective of these datasets must also be understood. For example, whilst many studies reported the diagnosis of a complication, this was not always paired with information on the treatment that the patient subsequently went on to receive. Re-operations not classified as re-revisions were poorly reported and so were not summarised. We recognise that elective, aseptic rKA is an ‘umbrella term’ with heterogeneity in patients, indications for surgery, severity of the disease, and types of procedure. In the future, estimates for clinical outcomes should be tailored to these different groups. To aid future systematic reviews and meta-analyses, studies reporting on rKA would benefit from consensus on how causes of failure should be categorised. In the meantime, use of a hierarchical system may be beneficial [66].

## Conclusion

Higher-quality evidence is needed to support patients with the decision of whether to undergo elective, aseptic rKA. This should include studies comparing operative and non-operative management. Implant survivorship following elective, aseptic rKA was ~ 96% at 1 year, ~ 91% at 5 years and ~ 87% at 10 years, with most studies identifying large improvements in pain and joint function. Early complications were common after elective, aseptic rKA and the rates summarised here can be shared with patients during informed consent.

## Supplementary Information

**Additional file 1:** **Appendix 1.** Search strategies. **Appendix 2. **Implant survivorship. **Appendix 2 Table 1.** Studies reporting implant survivorship following rKA using Kaplan-Meier estimates. **Appendix 2 Table 2.** Studies reporting implant survivorship for rKA expressed as person-time incidence rates (PTIR). **Appendix 2 Figure 1.** Forest plot of estimates for reported survival of revision KA (sensitivity analysis, where studies with missing 95% confidence intervals around Kaplan Meier estimates were excluded). **Appendix 3** – Patient-reported outcome measures. **Appendix 3 Table 1.** Studies reporting on PROM instruments. **Appendix 4.** Complications. **Appendix 4 Table 1.** Blood transfusion after rKA. **Appendix 5.** Quality of the included studies. **Appendix 5 Table 1.** Assessment of the methodological quality of the included studies using the checklist developed by Wylde et al for studies on joint arthroplasty.

## Data Availability

All available data is provided within the manuscript and supplementary files.
