# Supplementary material for: Patient-relevant outcomes following elective, aseptic revision knee arthroplasty: a systematic review
Source: Syst Rev. 2023 Aug 1;12:133. doi: 10.1186/s13643-023-02290-6 (PMC10394899; doi:10.1186/s13643-023-02290-6)
Supplement: Supplementary file 1 — Additional file 1: Appendix 1. Search strategies. Appendix 2. Implant survivorship. Appendix 2 Table 1. Studies reporting implant survivorship following rKA using Kaplan-Meier estimates. Appendix 2 Table 2. Studies reporting implant survivorship for rKA expressed as person-time incidence rates (PTIR). Appendix 2 Figure 1. Forest plot of estimates for reported survival of revision KA (sensitivity analysis, where studies with missing 95% confidence intervals around Kaplan Meier estimates were excluded). Appendix 3 – Patient-reported outcome measures. Appendix 3 Table 1. Studies reporting on PROM instruments. Appendix 4. Complications. Appendix 4 Table 1. Blood transfusion after rKA. Appendix 5. Quality of the included studies. Appendix 5 Table 1. Assessment of the methodological quality of the included studies using the checklist developed by Wylde et al for studies on joint arthroplasty. [file 13643_2023_2290_MOESM1_ESM.docx]

**Appendix 1 – Search strategies**

1. *Ovid Medline*

*Arthroplasty, Replacement, Knee/*

*((arthroplast* or replacement* or resurface*) adj3 knee*).ti,ab.*

*Knee Prosthesis/*

*((prosthes* or implant*) adj3 knee*).ti,ab.*

*(tka or tkr or ukr or uka).ti,ab.*

*1 or 2 or 3 or 4 or 5*

*revision*.ti,ab.*

*modular exchange*.ti,ab.*

*Reoperation/*

*(reoperation or re-operation or "repeat surg*").ti,ab.*

*7 or 8 or 9 or 10*

*6 and 11*

*Treatment Outcome/*

*outcome*.ti,ab.*

*Patient Reported Outcome Measures/*

*patient reported outcome measure*.ti,ab.*

*PROMs.ti,ab.*

*Comorbidity/*

*comorbid*.ti,ab.*

*13 or 14 or 15 or 16 or 17 or 18 or 19*

*12 and 20*

*randomized controlled trial.pt.*

*controlled clinical trial.pt.*

*randomized.ab.*

*randomly.ab.*

*trial.ab.*

*group*.ab.*

*22 or 23 or 24 or 25 or 26 or 27*

*exp Cohort Studies/*

*(cohort adj (study or studies)).tw.*

*(Follow up adj (study or studies)).tw.*

*(observational adj (study or studies)).tw.*

*Longitudinal.tw.*

*Retrospective.tw.*

*Prospective.tw.*

*29 or 30 or 31 or 32 or 33 or 34 or 35*

*28 or 36*

*21 and 37*

1. Embase

*knee arthroplasty/ or total knee arthroplasty/*

*((arthroplast* or replacement* or resurface*) adj3 knee*).ti,ab.*

*Knee Prosthesis/*

*((prosthe* or implant*) adj3 knee*).ti,ab.*

*(tka or tkr or ukr or uka).ti,ab.*

*1 or 2 or 3 or 4 or 5*

*revision*.ti,ab.*

*modular exchange*.ti,ab.*

*revision arthroplasty/*

*(reoperation or re-operation or "repeat surg*").ti,ab.*

*7 or 8 or 9 or 10*

*6 and 11*

*Treatment Outcome/*

*outcome*.ti,ab.*

*patient-reported outcome/*

*patient reported outcome measure*.ti,ab.*

*PROMs.ti,ab.*

*Comorbidity/*

*comorbid*.ti,ab.*

*13 or 14 or 15 or 16 or 17 or 18 or 19*

*12 and 20*

*randomized controlled trial/*

*single blind procedure/ or double blind procedure/*

*crossover procedure/*

*random*.ab.*

*trial.ab.*

*(random or ((singl* or doubl*) adj (blind* or mask*)) or crossover or cross over or factorial* or latin square or assign* or allocat* or volunteer*).ti,ab.*

*22 or 23 or 24 or 25 or 26 or 27*

*Cohort analysis/*

*(cohort adj (study or studies)).tw.*

*(Follow up adj (study or studies)).tw.*

*(observational adj (study or studies)).tw.*

*Longitudinal.tw.*

*Retrospective.tw.*

*Prospective.tw.*

*29 or 30 or 31 or 32 or 33 or 34 or 35*

*28 or 36*

*21 and 37*

**Appendix 2 – Implant survivorship**

*Appendix 2 Table 1 – Studies reporting implant survivorship following rKA using Kaplan-Meier estimates*

|  |  | **Implant Survivorship** | | |
| --- | --- | --- | --- | --- |
| **Study** | **No. knees***^1^* | **Estimate (%)** | **Lower confidence interval (%)** | **Upper confidence interval (%)** |
| **1 year** |  |  |  |  |
| Bloch et al (2020) | 316 | 99.7 | 97.8 | 100 |
| Sheng et al (2006)^3^ | 1874 | 95 | 94 | 96 |
| Sachdeva et al (2019)^3^ | 100 | 87 | NS | NS |
| Stockwell et al (2019) | 234 | 99.6 | 97.3 | 99.9 |
| Siqueira et al (2017)^2^ | 247 | 92.8 | NS | NS |
| Ong et al (2010)^2^ | 1599 | 94.1 | 92.7 | 95.1 |
| Bini et al (2016)^3^ | 1154 | 97.1 | 95.7 | 98.1 |
|  |  |  |  |  |
| **5 years** |  |  |  |  |
| Bloch et al (2020) | 316 | 98.7 | 96.5 | 99.5 |
| Sheng et al (2006) | 1874 | 89 | 88 | 90 |
| Stockwell et al (2019) | 234 | 92.3 | 87.9 | 95.2 |
| Hardeman et al (2011) | 146 | 90 | NS | NS |
| Liang et al (2017) | 258 | 97.8 | 97.1 | 99.1 |
| Leta et al (2016) | 308 | 91 | 87 | 94 |
| Engh et al (2012) | 119 | 87 | 81 | 93 |
| Stevens et al (2019) | 100 | 89 | 87.3 | 90.7 |
| Ong et al (2010)^2^ | 1599 | 87.4 | 85.2 | 89.3 |
| Siqueira et al (2017)^2^ | 247 | 81.3 | NS | NS |
| Kim et al (2015) | 194 | 100 | 94.3 | 100 |
| Wood et al (2009) | 135 | 95 | NS | NS |
| Bini et al (2016) | 1154 | 80 | 76 | 84 |
|  |  |  |  |  |
| **10 years** |  |  |  |  |
| Bloch et al (2020) | 316 | 97.8 | 94.2 | 99.2 |
| Sheng et al (2006) | 1874 | 79 | 78 | 81 |
| Siqueira et al (2017) | 247 | 75.8 | 70.4 | 81.7 |
| Kim et al (2015) | 194 | 97.8 | 92.5 | 99 |
| Suarez et al (2008)^4^ | 443 | 85 | 79 | 91 |
| Hardeman et al (2011) | 146 | 84.6 | NS | NS |
| Liang et al (2017) | 258 | 91.4 | 89.3 | 94.3 |
| Leta et al (2016) | 308 | 87 | 82 | 91 |
| Wood et al (2009)^4^ | 135 | 87 | NS | NS |
|  |  |  |  |  |
| **15 years** |  |  |  |  |
| Kim et al (2015)^5^ | 194 | 87.3 | 81.3 | 96.4 |
| Liang et al (2017) | 258 | 80.5 | 76.6 | 85.6 |

*^1^ We have presented the number of knees enrolled at the beginning of each study, since few studies reported the number of participants at-risk at each follow-up timepoint.*

*^2^ Estimate extracted from figure.*

*^3^ Follow-up rounded down to timepoint from 2-year estimate.*

*^4^ Follow-up rounded down to timepoint from 12-year estimate.*

*^5^ Follow-up rounded down to timepoint from 16-year estimate.*

*NS – Not specified.*

*Appendix 2 Table 2 – Studies reporting implant survivorship for rKA expressed as person-time incidence rates (PTIR)*

| **Study^1^** | **Time period^2^** | **Mean follow-up (years)** | **No. knees** | **No. revisions** | **PTIR^3^** |
| --- | --- | --- | --- | --- | --- |
| Martin et al (2020) | Medium term | 3.5 | 164 | 12 | 2.1 |
| Graichen et al (2015) | Medium term | 3.6 | 121 | 14 | 3.2 |
| Turnbull et al (2019) | Medium term | 3.9 | 112 | 16 | 3.7 |
| Sachdeva et al (2019) | Medium term | 4.3 | 100 | 13 | 3.0 |
| Engh et al (2012) | Medium term | 4.6 | 119 | 17 | 3.1 |
| Hardeman et al (2011) | Medium term | 4.8 | 146 | 13 | 1.9 |
| Stockwell et al (2019) | Medium term | 4.9 | 234 | 16 | 1.4 |
| Kim et al (2017) | Medium term | 4.9 | 280 | 29 | 2.1 |
| Wood et al (2009) | Medium term | 5 | 135 | 6 | 0.9 |
| Crawford et al (2017) | Long term | 6 | 278 | 25 | 1.5 |
| Lombardi et al (2018) | Long term | 6.1 | 193 | 13 | 1.1 |
| Sierra et al (2013) | Long term | 6.3 | 175 | 9 | 0.8 |
| Bugbee et al (2001) | Long term | 7 | 123 | 20 | 2.3 |
| Stevens et al (2019) | Long term | 7.2 | 100 | 12 | 1.7 |
| Bloch et al (2020) | Long term | 7.6 | 316 | 5 | 0.2 |
| Bin et al (2019) | Long term | 8.4 | 163 | 1 | 0.1 |
| Liang et al (2017) | Long term | 9.8 | 258 | 21 | 0.8 |
| Kim et al (2015) | Long term | 14.6 | 194 | 18 | 0.6 |

*^1^Sorted by mean follow-up time from revision KA*

*^2^Medium term defined as 1-5 years; Long-term defined as >5 years*

*^3^PTIR = Person time incidence rate of re-revision KA per 100 person-years*

*Appendix 2 Figure 1 – Forest plot of estimates for reported survival of revision KA (sensitivity analysis, where studies with missing 95% confidence intervals around Kaplan Meier estimates were excluded)*


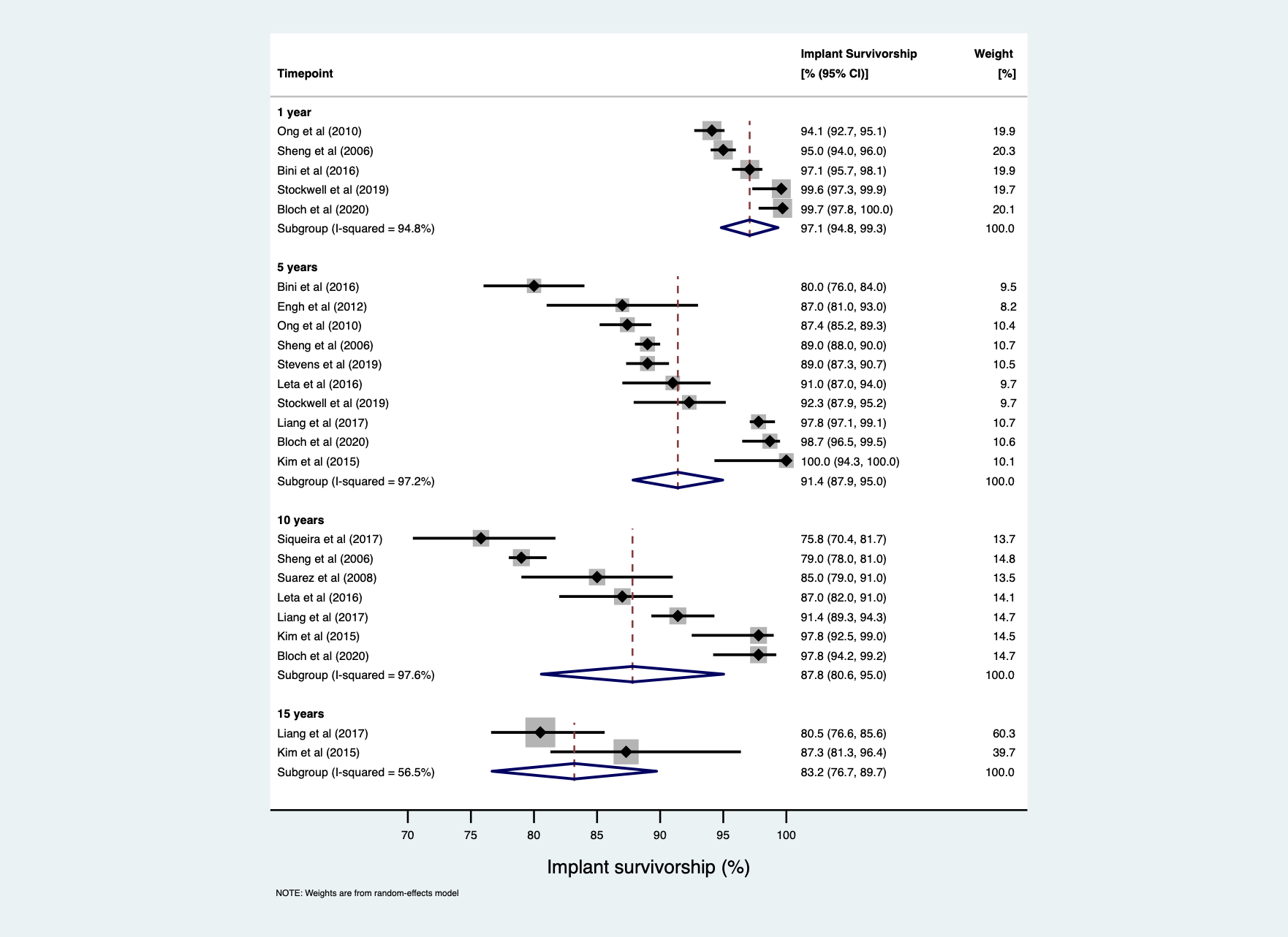


**Appendix 3 – Patient-reported outcome measures**

*Appendix 3 Table 1 – Studies reporting on PROM instruments*

| **Instrument** | **Subscale** | **Study ^1^** | **Study Design** | **Timepoint** | **No. rKA ^2^** | **Scale (Best/Worst)** | **Pre-operative score (mean [sd])** | **Post-operative score (mean [sd])** | **Change in score (mean [sd])** |
| --- | --- | --- | --- | --- | --- | --- | --- | --- | --- |
| ***Joint-specific*** |  |  |  |  |  |  |  |  |  |
| *KOOS* |  |  |  |  |  |  |  |  |  |
|  | *ADL* | Leta et al (2016) | Retrospective observational (NAR) | "At least 1 year" | 114 | 100/0 | NS | 52 [24] | NS |
|  | *Pain* | Piuzzi et al (2020) | Prospective cohort (OME) | 1 year | 246 | 100/0 | 39.9 [19.9] ^3^ | NS | 30.3 [24.5] |
|  | *Pain* | Leta et al (2016) | Retrospective observational (NAR) | "At least 1 year" | 114 | 100/0 | NS | 55 [25] | NS |
|  | *PS* | Piuzzi et al (2020) | Prospective cohort (OME) | 1 year | 246 | 100/0 | 45.9 [17.8] ^3^ | NS | 19.2 [22.5] |
|  | *QoL* | Piuzzi et al (2020) | Prospective cohort (OME) | 1 year | 246 | 100/0 | 18.5 [18.8] ^3^ | NS | 29.7 [28.0] |
|  | *QoL* | Leta et al (2016) | Retrospective observational (NAR) | "At least 1 year" | 114 | 100/0 | NS | 38 [26] | NS |
|  | *Symptoms* | Leta et al (2016) | Retrospective observational (NAR) | "At least 1 year" | 114 | 100/0 | NS | 64 [20] | NS |
|  | *Sports & Recreation* | Leta et al (2016) | Retrospective observational (NAR) | "At least 1 year" | 114 | 100/0 | NS | 17 [22] | NS |
|  |  |  |  |  |  |  |  |  |  |
| *OKS* |  |  |  |  |  |  |  |  |  |
|  |  | Baker et al (2012) | Retrospective observational (NJR-PROMs) | 6 months | 797 | 48/0 | 16.2 [8.6] | 26.6 [11.5] | 10.4 [10.1] |
|  |  | Stockwell et al (2019) | Retrospective observational (single centre) | 1 year | 209 | 48/0 | 18.8 [NS] | 31.7 [NS] | NS |
|  |  | Stockwell et al (2019) | Retrospective observational (single centre) | 2 years | 170 | 48/0 | 18.8 [NS] | 30.7 [NS] | NS |
|  |  | Bin Abd Razak et al (2019) ^4^ | Retrospective observational (single centre) | 2 years | 163 | 48/0 | 21 [NS] | 36 [NS] | 15 [NS] |
|  |  | Stockwell et al (2019) | Retrospective observational (single centre) | "Last follow-up" (mean 5.1 years) | 139 | 48/0 | 18.8 [NS] | 30.6 [NS] | NS |
|  |  | Turnbull et al (2019) | Retrospective observational (single centre) | "Last follow-up" (mean 3.9 years) | 112 | 48/0 | 15 [8.8] | 27 [11.1] | NS |
|  |  | Bin Abd Razak et al (2019) ^4^ | Retrospective observational (single centre) | 5 years | 163 | 48/0 | 21 [NS] | 38 [NS] | 17 [NS] |
|  |  | Stevens et al (2019) | Retrospective observational (single centre) | "Minimum 5 year, median 7.2 years" | 45 | 48/0 | NS | 27 [11.9] | NS |
|  |  |  |  |  |  |  |  |  |  |
| *WOMAC* |  |  |  |  |  |  |  |  |  |
|  | *Pain* | Martin-Hernandez et al (2017) ^4^ | Prospective cohort (single centre) | 3 months | 134 | 100/0 | 40 [NS] | 55 [NS] | NS |
|  | *Pain* | Martin-Hernandez et al (2017) ^4^ | Prospective cohort (single centre) | 1 year | 134 | 100/0 | 40 [NS] | 75 [NS] | NS |
|  | *Pain* | Malviya et al (2012) | Retrospective observational (single centre) | 1 year | 120 | 100/0 | 34.5 [14.9] | 61.9 [19.1] | NS |
|  | *Pain* | Kasmire et al (2014) ^4^ | Retrospective observational (single centre) | 2 years | 175 | 100/0 | 48.5 [NS] | 74 [NS] | NS |
|  | *Pain* | Venkataramanan et al (2013) | Retrospective observational (multi-centre) | 2 years | 145 | 100/0 | 46.5 [19.8] | 71.3 [24.4] | NS |
|  | *Pain* | Martin-Hernandez et al (2017) ^4^ | Prospective cohort (single centre) | "Last follow-up" (median 71.5 months) | 134 | 100/0 | 40 [NS] | 80 [NS] | NS |
|  |  |  |  |  |  |  |  |  |  |
|  | *Stiffness* | Martin-Hernandez et al (2017) ^4^ | Prospective cohort (single centre) | 3 months | 134 | 100/0 | 37.5 [NS] | 62.5 [NS] | NS |
|  | *Stiffness* | Martin-Hernandez et al (2017) ^4^ | Prospective cohort (single centre) | 1 year | 134 | 100/0 | 37.5 [NS] | 75 [NS] | NS |
|  | *Stiffness* | Malviya et al (2012) | Retrospective observational (single centre) | 1 year | 120 | 100/0 | 40.4 [17.1] | 59.7 [18.7] | NS |
|  | *Stiffness* | Kasmire et al (2014) ^4^ | Retrospective observational (single centre) | 2 years | 175 | 100/0 | 47.5 [NS] | 68.8 [NS] | NS |
|  | *Stiffness* | Martin-Hernandez et al (2017) ^4^ | Prospective cohort (single centre) | "Last follow-up" (median 71.5 months) | 134 | 100/0 | 37.5 [NS] | 75 [NS] | NS |
|  |  |  |  |  |  |  |  |  |  |
|  | *Function* | Martin-Hernandez et al (2017) ^4^ | Prospective cohort (single centre) | 3 months | 134 | 100/0 | 33.8 [NS] | 51.5 [NS] | NS |
|  | *Function* | Martin-Hernandez et al (2017) ^4^ | Prospective cohort (single centre) | 1 year | 134 | 100/0 | 33.8 [NS] | 73.5 [NS] | NS |
|  | *Function* | Malviya et al (2012) | Retrospective observational (single centre) | 1 year | 120 | 100/0 | 32.1 [16.6] | 54.6 [20.1] | NS |
|  | *Function* | Kasmire et al (2014) ^4^ | Retrospective observational (single centre) | 2 years | 175 | 100/0 | 49.7 [NS] | 70.0 [NS] | NS |
|  | *Function* | Venkataramanan et al (2013) | Retrospective observational (multi-centre) | 2 years | 145 | 100/0 | 45.5 [20] | 65.8 [22.3] | NS |
|  | *Function* | Martin-Hernandez et al (2017) ^4^ | Prospective cohort (single centre) | "Last follow-up" (median 71.5 months) | 134 | 100/0 | 33.8 [NS] | 79.4 [NS] | NS |
|  |  |  |  |  |  |  |  |  |  |
|  | *Total* | Kim et al (2015) ^4^ | Retrospective observational (single centre) | 1 year | 192 | 100/0 | 8.3 [NS] | 74.0 [NS] | NS |
|  | *Total* | Kim et al (2015) ^4^ | Retrospective observational (single centre) | 5 years | 183 | 100/0 | 8.3 [NS] | 75.0 [NS] | NS |
|  | *Total* | Kim et al (2015) ^4^ | Retrospective observational (single centre) | 10 years | 183 | 100/0 | 8.3 [NS] | 76.0 [NS] | NS |
|  | *Total* | Kim et al (2015) ^4^ | Retrospective observational (single centre) | 15 years | 183 | 100/0 | 8.3 [NS] | 74.0 [NS] | NS |
|  |  |  |  |  |  |  |  |  |  |
| ***Generic*** |  |  |  |  |  |  |  |  |  |
| *EQ-5D utility* |  |  |  |  |  |  |  |  |  |
|  |  | Baker et al (2012) ^5^ | Retrospective observational (NJR-PROMs) | 6 months | 797 | 1.00 to -0.59 | 0.310 [0.346] | 0.541 [0.382] | 0.231 [0.338] |
|  |  | Leta et al (2016) | Retrospective observational (NAR) | "At least 1 year" | 114 | 1.00 to -0.59 | 0.41 [0.21] | 0.56 [0.25] | NS |
|  |  |  |  |  |  |  |  |  |  |
| *SF-12* |  |  |  |  |  |  |  |  |  |
|  | *PCS* | Martin-Hernandez et al (2017) | Prospective cohort (single centre) | 3 months | 134 | 100/0 | 27 [NS] | 37 [NS] | NS |
|  | *PCS* | Martin-Hernandez et al (2017) | Prospective cohort (single centre) | 1 year | 134 | 100/0 | 27 [NS] | 41 [NS] | NS |
|  | *PCS* | Martin-Hernandez et al (2017) | Prospective cohort (single centre) | "Last follow-up" (median 71.5 months) | 134 | 100/0 | 27 [NS] | 44 [NS] | NS |
|  | *PCS* | Stevens et al (2019) | Retrospective observational (single centre) | "Minimum 5 year, median 7.2 years" | 45 | 100/0 | NS | 40.6 [17.6] | NS |
|  |  |  |  |  |  |  |  |  |  |
|  | *MCS* | Martin-Hernandez et al (2017) | Prospective cohort (single centre) | 3 months | 134 | 100/0 | 43 [NS] | 48 [NS] | NS |
|  | *MCS* | Martin-Hernandez et al (2017) | Prospective cohort (single centre) | 1 year | 134 | 100/0 | 43 [NS] | 51 [NS] | NS |
|  | *MCS* | Martin-Hernandez et al (2017) | Prospective cohort (single centre) | "Last follow-up" (median 71.5 months) | 134 | 100/0 | 43 [NS] | 54 [NS] | NS |
|  | *MCS* | Stevens et al (2019) | Retrospective observational (single centre) | "Minimum 5 year, median 7.2 years" | 45 | 100/0 | NS | 48.3 [15.5] | NS |
|  |  |  |  |  |  |  |  |  |  |
| *SF-36* |  |  |  |  |  |  |  |  |  |
|  | *PCS* | Kasmire et al (2014) | Retrospective observational (single centre) | 2 years | 175 | 100/0 | 40.7 [NS] | 55.5 [NS] | NS |
|  | *PCS* | Bin Abd Razak et al (2019) | Retrospective observational (single centre) | 2 years | 163 | 100/0 | 28 [NS] | 45 [NS] | 17 [NS] |
|  | *PCS* | Bin Abd Razak et al (2019) | Retrospective observational (single centre) | 5 years | 163 | 100/0 | 28 [NS] | 46 [NS] | 18 [NS] |
|  |  |  |  |  |  |  |  |  |  |
|  | *MCS* | Kasmire et al (2014) | Retrospective observational (single centre) | 2 years | 175 | 100/0 | 60.3 [NS] | 70.2 [NS] | NS |
|  | *MCS* | Bin Abd Razak et al (2019) | Retrospective observational (single centre) | 2 years | 163 | 100/0 | 49 [NS] | 52 [NS] | 3 [NS] |
|  | *MCS* | Venkataramanan et al (2013) | Retrospective observational (multi-centre) | 2 years | 145 | 100/0 | 51.9 [11.5] | 54.6 [9.7] | NS |
|  | *MCS* | Bin Abd Razak et al (2019) | Retrospective observational (single centre) | 5 years | 163 | 100/0 | 49 [NS] | 53 [NS] | 4 [NS] |
|  |  |  |  |  |  |  |  |  |  |

*^1^ Sorted by PROM instrument, subscale, timepoint then study size*

*^2^ rKA who responded to PROM questionnaire*

*^3^ Cohort with 1-year PROM available*

*^4^ Reported scores transformed to the scale indicated*

*^5^ Standard deviations calculated from 95% confidence intervals*

*ADL - Activities of daily living*

*KOOS - Knee Injury and Osteoarthritis Outcome Score*

*MCS - Mental component score*

*NAR - Norwegian Arthroplasty Register*

*NJR - National Joint Registry*

*NS - Not specified*

*OKS - Oxford Knee Score*

*OME - Orthopaedic Minimal Data Set Episode of Care database*

*PCS - Physical component score*

*PROMs - NHS Patient Reported Outcome Measures*

*PS - KOOS Physical Function Short Form*

*QoL - Quality of life*

*SF - Short form*

*WOMAC - Western Ontario and McMaster Universities Arthritis Index*

**Appendix 4 - Complications**

*Appendix 4 Table 1 – Blood transfusion after rKA*

| **Study ^1^** | **Study Design** | **Timepoint** | **No. rKA** | **Transfused**  **n (%)** |
| --- | --- | --- | --- | --- |
| Nichols et al (2016) | Retrospective observational (MarketScan) | Immediate  (“in hospital”) | 25354 | 2130 (8.4) |
| Burnett et al (2017) | Retrospective observational (Humana Inc) | Immediate  (3 days) | 12493 | 1482 (11.9) |
| Boddapati et al (2017) | Retrospective observational (NSQIP) | Immediate  (30 days) | 10584 | 1256 (11.9) |
| Dai et al (2020) | Retrospective observational (NIS) | Immediate  (“in hospital”) | 5187 | 955 (18.4) |

*^1^ Sorted by study size*

*NIS - Nationwide Inpatient Sample*

*NSQIP - American College of Surgeons National Surgical Quality Improvement Program*

**Appendix 5**

*Appendix 5 Table 1 – Assessment of the methodological quality of the included studies using the checklist developed by Wylde et al for studies on joint arthroplasty*

| **Author** | **Consecutive patients** | **Representativeness^1^** | **Follow-up^2^** | **Minimisation of confounding^3^** |
| --- | --- | --- | --- | --- |
| Baker et al (2012) | - | + | - | - |
| Bin Abd Razak et al (2019) | + | - | - | - |
| Bini et al (2016) | - | + | + | + |
| Bloch et al (2020) | - | - | + | - |
| Boddapati et al (2018) | - | + | + | + |
| Boylan et al (2017) | - | + | + | + |
| Bugbee et al (2001) | + | - | + | - |
| Burnett et al (2017) | - | + | + | - |
| Crawford et al (2017) | + | - | - | - |
| Dai et al (2020) | - | + | + | + |
| Edmiston et al (2019) | - | + | + | + |
| Engh et al (2012) | - | - | + | - |
| Graichen et al (2015) | + | - | + | - |
| Hardeman et al (2012) | + | - | + | - |
| Kasmire et al (2014) | - | - | + | + |
| Kim et al (2017) | + | + | + | - |
| Kim et al (2015) | - | - | + | - |
| Kremers et al (2014) | - | - | + | + |
| Leta et al (2016) | - | + | - | + |
| Liang et al (2017) | + | - | - | + |
| Lombardi et al (2018) | + | - | + | - |
| Malviya et al (2012) | + | - | + | + |
| Martin et al (2020) | + | - | - | - |
| Martin-Hernandez et al (2017) | + | - | + | - |
| Memtsoudis et al (2008) | + | + | + | + |
| Nichols et al (2016) | - | + | + | + |
| Ong et al (2010) | - | + | + | + |
| Piuzzi et al (2020) | - | - | - | + |
| Sachdeva et al (2019) | - | - | + | - |
| Schairer et al (2014) | + | - | + | + |
| Sheng et al (2006) | + | + | + | + |
| Sierra et al (2013) | + | + | + | - |
| Siqueira et al (2017) | + | - | + | + |
| Stevens et al (2019) | + | - | + | - |
| Stockwell et al (2019) | + | - | + | - |
| Suarez et al (2008) | + | - | + | - |
| Turnbull et al (2019) | - | - | - | + |
| Venkataramanan et al (2013) | - | + | - | + |
| Wood et al (2009) | + | - | + | - |
| Yao et al (2019) | + | - | + | + |

*+ (adequate), - (inadequate)*

*^1^ Multicentre studies rated ‘+’*

*^2^ A follow-up rate greater than 80% rated ‘+’*

*^3^ Use of multivariate analysis rated ‘+’*
